# Molecular Beam Epitaxial Growth and Optical Properties of InN Nanostructures on Large Lattice-Mismatched Substrates

**DOI:** 10.3390/ma17246181

**Published:** 2024-12-18

**Authors:** Rongtao Nie, Yifan Hu, Guoguang Wu, Yapeng Li, Yutong Chen, Haoxin Nie, Xiaoqiu Wang, Mengmeng Ren, Guoxing Li, Yuantao Zhang, Baolin Zhang

**Affiliations:** State Key Laboratory of Integrated Optoelectronics, College of Electronic Science and Engineering, Jilin University, Changchun 130012, China; niert22@mails.jlu.edu.cn (R.N.); huyf21@mails.jlu.edu.cn (Y.H.); ypli24@mails.jlu.edu.cn (Y.L.); chenyt22@mails.jlu.edu.cn (Y.C.); niehx23@mails.jlu.edu.cn (H.N.); wangxq22@mails.jlu.edu.cn (X.W.); renmm23@mails.jlu.edu.cn (M.R.); zbl@jlu.edu.cn (B.Z.)

**Keywords:** near-infrared, InN, nanostructure, liquid-phase epitaxial, temperature stability

## Abstract

Narrow-gap InN is a desirable candidate for near-infrared (NIR) optical communication applications. However, the absence of lattice-matched substrates impedes the fabrication of high-quality InN. In this paper, we employed Molecular Beam Epitaxy (MBE) to grow nanostructured InN with distinct growth mechanisms. Morphological and quality analysis showed that the liquid phase epitaxial (LPE) growth of hexagonal InN nanopillar could be realized by depositing molten In layer on large lattice-mismatched sapphire substrate; nevertheless, InN nanonetworks were formed on nitrided sapphire and GaN substrates through the vapor-solid process under the same conditions. The supersaturated precipitation of InN grains from the molten In layer effectively reduced the defects caused by lattice mismatch and suppressed the introduction of non-stoichiometric metal In in the epitaxial InN. Photoluminescence and electrical characterizations demonstrated that high-carrier concentration InN prepared by vapor-solid mechanism showed much stronger band-filling effect at room temperature, which significantly shifted its PL peak to higher energy. LPE InN displayed the strongest PL intensity and the smallest wavelength shift with increasing temperature from 10 K to 300 K. These results showed enhanced optical properties of InN nanostructures prepared on large lattice mismatch substrates, which will play a crucial role in near-infrared optoelectronic devices.

## 1. Introduction

Recent research has identified two principal challenges for InGaAsP-based NIR light-emitting devices, increasing surface and sidewall recombination rates and the sensitivity of the emission wavelength to temperature variations, which adversely affect the stability and performance of the device [1,2,3,4]. InN within the III-V nitrides is recognized for its superior electron saturation velocity and drift velocity. Its narrow direct bandgap of approximately 0.70 eV aligns with the near-infrared spectrum, coupled with high quantum efficiency and excellent thermal stability within the thermal range of 10 to 300 K, positioning it as a prospective material for high-speed near-infrared optical communication applications [5,6,7,8,9,10,11]. Notably, the effective surface strain relaxation of InN nanostructures can suppress the generation of dislocations, and its quantum confinement effect limits carrier diffusion, which helps to reduce the surface recombination rate and non-radiative recombination [12,13,14,15]. Moreover, the significant specific surface area of the InN nanostructure significantly enhances the interaction between incident light and InN materials and helps to improve light absorption and luminescence efficiency. And in recent years, researchers have conducted comprehensive investigations on the growth mechanisms of III-V nitride nanostructures [16,17]. However, the synthesis of high-quality InN has always been a significant challenge. It is mainly due to the lack of lattice-matching substrate and lower decomposition temperature (600 °C) of InN during heteroepitaxy, which leads to the formation of dislocations during low-temperature growth and thus reduces the electrical and optical properties [18].

Some researchers have employed different substrates to reduce the influence of lattice mismatch. Matthieu et al. [19] employed metal-organic vapor phase epitaxy (MOVPE) to nitride the surface of sapphire substrates with different orientations to form several nanometers of the AlN (13% lattice mismatch) thin layer, which is beneficial to enhance the lattice matching and reduce the defect density in InN. Ali et al. [5] utilized GaN (9% lattice mismatch) substrate to prepare InN/GaN heterostructures for application in high-performance broadband photodetectors. Malleswararao et al. [20] used GaN nanowall network templates to prevent dislocation propagation across the InN/GaN interface. While high-quality InN can be achieved through epitaxy on GaN substrates, the high cost of GaN and the specialized treatments required for the substrates pose significant economic challenges. Furthermore, the growth of III-V nitride nanostructure induced by the catalysis of pre-deposited specific metal nanoparticles has also been studied [21,22,23]. A similar LPE process was adopted to GaN crystal growth [24]. Huang et al. [25] achieved high-quality GaN crystal growth by modulating the supersaturation of GaN in the Ga-Na melt under high-temperature and pressure conditions (850 °C, 3 MPa). In the same year, Katsuumi et al. [26] substituted the commonly used nitrogen source (N_2_) with iron nitride (Fe_3_N), which allowed for the preparation conditions to shift from high pressure to atmospheric pressure. However, the introduction of Fe_3_N resulted in the reduction in GaN crystal quality. In contrast, Moustakas et al. [27] demonstrated a novel approach to LPE of GaN through MBE, which differs from the traditional high-temperature and high-pressure LPE methods. Notably, contemporary reports on the epitaxial growth of InN using MBE with LPE mechanism are scarce. This method allows for more efficient dissolution of reactive nitrogen energy in the liquid-phase metal In, avoiding the hazardous conditions associated with high pressure. Additionally, the lower growth temperature (480 °C) in the liquid-phase In environment minimizes the introduction of carbon-oxidized impurities compared to vapor-solid mechanism growth [27].

In this study, we report on the comparative analysis of the MBE technique employing a pre-deposited around 40 nm thick film layer of molten metallic In on sapphire substrates, contrasted with two conventional vapor-solid methods: the epitaxial growth of InN on nitrided sapphire substrate and sapphire-based GaN substrate. This study aims to investigate the impact of different growth mechanisms on the properties of InN.

## 2. Materials and Methods

**Figure 1:**
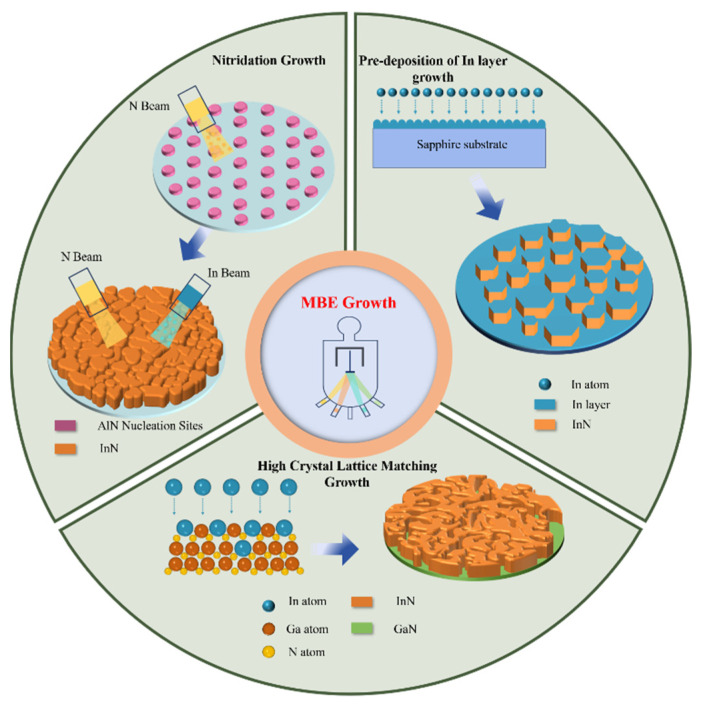
Three different sets of substrate treatment and epitaxy methods. The top left inset illustrates the growth of InN on nitrided sapphire substrate, the top right inset depicts the growth of InN on sapphire substrate with pre-deposited molten In layer, and the bottom inset presents the direct growth of InN on GaN substrate.

We utilized MBE to fabricate three distinct InN samples on nitrided c-face sapphire substrates, GaN substrates, and sapphire substrates pre-deposited with metallic In layers, as depicted in Figure 1. The specific preparation parameters are as follows:

For sample A, the sapphire substrate underwent thermal cleaning, followed by nitridation at 500 °C for 1 h with a N_2_ flow rate of 1.5 sccm and a nitrogen plasma power of 400 W, aimed at forming a thin AlN layer to reduce lattice mismatch with epitaxial InN. Subsequently, the two-step growth process was employed: Initially, a low-temperature buffer layer was formed at 400 °C with a N_2_ flow rate of 2 sccm, nitrogen plasma power of 400 W, indium source temperature of 660 °C, and a growth duration of 50 min. Subsequently, the high-temperature epitaxial layer growth stage commenced with the substrate temperature increased to 480 °C, maintaining other parameters, and a growth duration of 170 min. For sample B, InN was directly epitaxialized on GaN substrate employing the same two-step process parameters as described for sapphire nitride. For sample C, when post-thermal cleaning the sapphire substrate, around a 40 nm thick indium layer was pre-deposited at 450 °C, followed by growth using the same two-step parameters as detailed for nitrided sapphire.

The surface structure and growth kinetics of the substrates, pre-treated surfaces, and epitaxial InN were characterized using reflectance high-energy electron diffraction (RHEED, CREATEC/PR-MBE, Erligheim, Germany), while scanning electron microscopy (SEM, JEOL/JSM-7500F, Tokyo, Japan) was employed to analyze the surface morphology of the samples. Crystal quality was assessed through X-ray diffraction (XRD, Rigaku/Ultima IV, Tokyo, Japan) analysis. Surface composition was determined using X-ray photoelectron spectroscopy (XPS, CREATEC/PR-MBE, Erligheim, Germany). Optical properties were assessed through photoluminescence (PL) spectroscopy; a 514 nm Argon ion laser served as the excitation source, coupled with a liquid nitrogen-cooled germanium detector. A liquid helium cooling system was utilized to perform PL measurements over the temperature range of 10 to 300 K.

## 3. Results and Discussion

**Figure 2:**
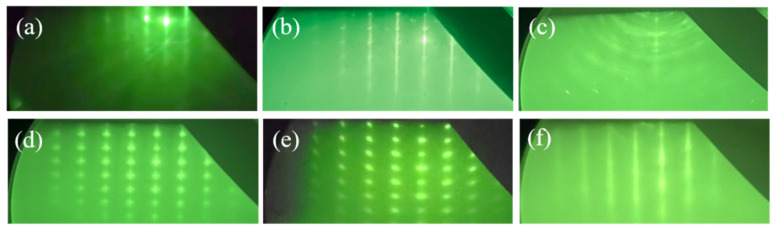
RHEED patterns of (**a**) nitrided C-face sapphire substrate. (**b**) GaN substrate. (**c**) Sapphire substrate pre-deposited with around 40 nm molten metal In. (**d**) Sample A: InN grew on a nitrided sapphire substrate. (**e**) Sample B: InN grown directly on GaN substrate. (**f**) Sample C: InN grown on sapphire substrate with pre-deposited In metal.

Figure 2 presents RHEED patterns of nitrided sapphire substrate, a GaN substrate with low lattice mismatch, a sapphire substrate with a pre-deposited metallic In layer, and epitaxially grown InN on these substrates under identical conditions. As shown in Figure 2a, the RHEED pattern of the nitrided sapphire substrate displays streaks. These findings indicate that nitrogen bombardment during nitridation effectively removes surface-adsorbed impurities and dangling bonds (e.g., Al-OH). Concurrently, nitrogen atom bombardment reduces Al-O_x_ bond content, with nitrogen atoms partially substituting oxygen on the surface, thereby reducing the lattice mismatch between the substrate and InN [28]. Figure 2b presents the RHEED patterns for GaN substrate, which exhibit clear and regular diffraction streaks, indicative of a flat and smooth single-crystalline GaN film. Post-deposition of the In metal layer on the sapphire substrate, the RHEED pattern in Figure 2c displays a diffuse ring and irregular dot pattern, suggesting complete coverage of the sapphire substrate by the amorphous In metal layer. Comparative analysis of the RHEED patterns for InN grown on the three distinct substrates reveals that the epitaxial InN layers in Figure 2d,e both display regular dot-like array diffraction patterns, signifying the formation of nanostructured, single-crystalline InN epitaxial layers in both instances. Figure 2f displays a distinct diffraction pattern, characterized by a bright, sharp combination of stripes and dots. These observations suggest that, despite the poor lattice matching of the pre-deposited metallic In layer, this growth method still produces high-quality single-crystalline InN layers with a smoother, flatter crystal surface and excellent orientation uniformity compared to the InN nano-monocrystals formed by samples A and B. This is noteworthy given that the pre-deposited metallic In layer does not align well with the lattice, highlighting the capacity of epitaxial growth to achieve high-quality InN layers despite lattice mismatch challenges.

**Figure 3:**
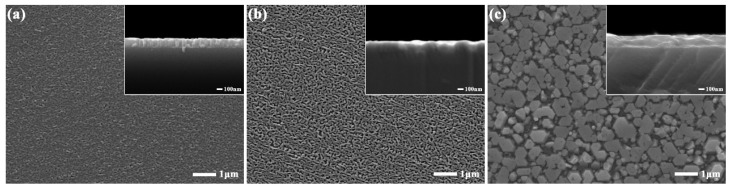
SEM images showing surface and cross-sectional view of sample A (**a**), sample B (**b**), and sample C (**c**).

Figure 3 elucidates the surface and cross-sectional topography of epitaxial InN on three distinct substrates. The InN layers in Figure 3a,b manifest a nano-network structure, which is responsible for the dot-like RHEED diffraction patterns observed in Figure 2a,b. Notably, the InN epitaxial layer in Figure 3a exhibits smaller reticulated surface micropores and thicker nanowalls compared to Figure 3b. This is attributed to the higher nucleation site density on the nitrided sapphire substrate, promoting enhanced lateral growth. The flat and smooth GaN substrate surface exhibits a lower density of nucleation sites and a higher rate of longitudinal growth, as evidenced by the increased thickness of the InN epitaxial layer in its cross-sectional view. Figure 3c illustrates the InN epitaxial layer obtained following the deposition of an In layer on the sapphire substrate. This morphology is markedly distinct, featuring a hexagonal nanopillar structure with a column diameter of approximately 400 nm and a significantly smoother and flatter surface compared to the other samples. Additionally, the nanopillars exhibit a more uniform rotation angle. The results indicate that when MBE is utilized for the epitaxial growth of InN on nitrided sapphire and GaN substrates, the growth mechanism is predominantly the conventional vapor-solid method. InN crystals are formed through the interaction of In and N molecular beams at dispersed nucleation sites on the substrate surface. The overall morphology of the epitaxial InN depends on the lattice structure of the substrate. Upon the deposition of the molten metal layer on the sapphire substrate, the In and N molecular beam currents impinge on the liquid-phase In, precipitating InN crystals through a maturation process. As the grains grow, further adsorption of the reactive beam current occurs; however, growth competition among the grains leads to the coexistence of nanopillars with diameters up to 400 nm alongside smaller nanopillars on the surface of the final substrate, leading to the absence of lateral grain bridging observed in Figure 3a,b, characteristic of a lateral linkage structure. Moreover, the epitaxial growth by LPE minimally perturbs the grain morphology beyond the In layer, despite the underlying sapphire substrate’s lattice structure. However, the hexagonal zincite crystal structure exerts a pronounced influence, directing the formation of regular hexagonal InN pillars.

**Figure 4:**
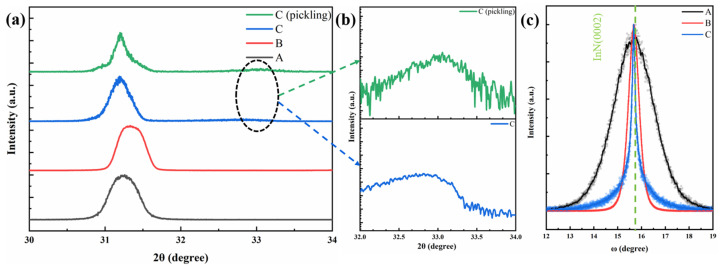
(**a**) Normalized XRD patterns of samples A, B, and C and pickled sample C; (**b**) XRD patterns with logarithmic vertical scaling; (**c**) the normalized ω scans of InN (002) derived from different growth methods.

Figure 4 presents the XRD analysis results for the three InN samples, A, B, and C. As shown in Figure 4a, the InN (002) diffraction peak at approximately 31.3° is evident in the 2θ scans of all samples, indicating c-axis preferential orientation. A weak indium metal singlet diffraction peak is observed near 33° for sample C. To ascertain the origin of this In signal, sample C was treated with 5% hydrochloric acid for 5 min. The logarithmic plots of the In singlet diffraction peaks before and after treatment are shown in Figure 4b. The alteration in peak shape is not eliminated. These results suggest that the indium metal primarily originates from the previously pre-deposited In layer on the substrate surface. This implies that the indium metal mainly stems from the pre-deposited In layer on the sapphire surface, with a possible minor presence of In monomers on the InN epitaxial layer surface. The lattice constants of the three InN samples were determined using the Bragg equation, as outlined in Equation (1):

(1) 2dsin⁡θ=nλ ,

where *θ* represents the angle of incidence of the X-ray, d denotes the interplanar spacing, *λ* signifies the X-ray wavelength, and *n* is the order of diffraction. The results are tabulated in Table 1. With the c-axis lattice constant of an ideal InN single crystal being 0.5693 nm, the epitaxial samples A, B, and C exhibit compressive strains of 0.40%, 0.18%, and 0.58%, respectively. The higher compressive stress in sample A compared to B aligns with the lattice mismatch between the substrate and InN in these samples. Notably, sample C experiences the most significant compressive stress, attributable to the substantial difference in the thermal expansion coefficients of metallic In (~28.4 × 10^−6^ K^−1^) [29] and InN (~2.6 × 10^−6^ K^−1^) [30] during the cooling phase post-growth, leading to substantial compressive stress in the InN nanopillars of sample C. The ω-scan XRD patterns in Figure 4c reveal significant variations in the full width at half maxima (FWHM) of the rocking curves for the three samples. The helical dislocation densities within the InN crystals were calculated using Equation (2) [31]:

(2) $$\rho =\frac{\beta^{2}}{4.35\times b^{2}}\;$$

where *ρ* and *β* denote the helical dislocation density and the FWHM value of the rocking curve, respectively. The calculations, as detailed in Table 1, reveal that the dislocation density of epitaxial InN on the nitrided sapphire substrate exceeds that on the GaN substrate. This can be attributed to the vapor-solid growth phase of samples A and B. The stress calculations for epitaxial InN indicate that sample A experiences greater stress than sample B, leading to a higher dislocation density due to substrate lattice mismatch. Sample C, with its distinct LPE growth mode, exhibits a lower dislocation density during growth. Despite the elevated stress in the InN nanopillars of sample C due to thermal mismatch during cooling, this does not significantly increase the dislocation density within the nanopillars. Consequently, the epitaxial InN nanopillars in sample C show the lowest dislocation density following the deposition of metallic In on the sapphire substrate.

**Figure 5:**
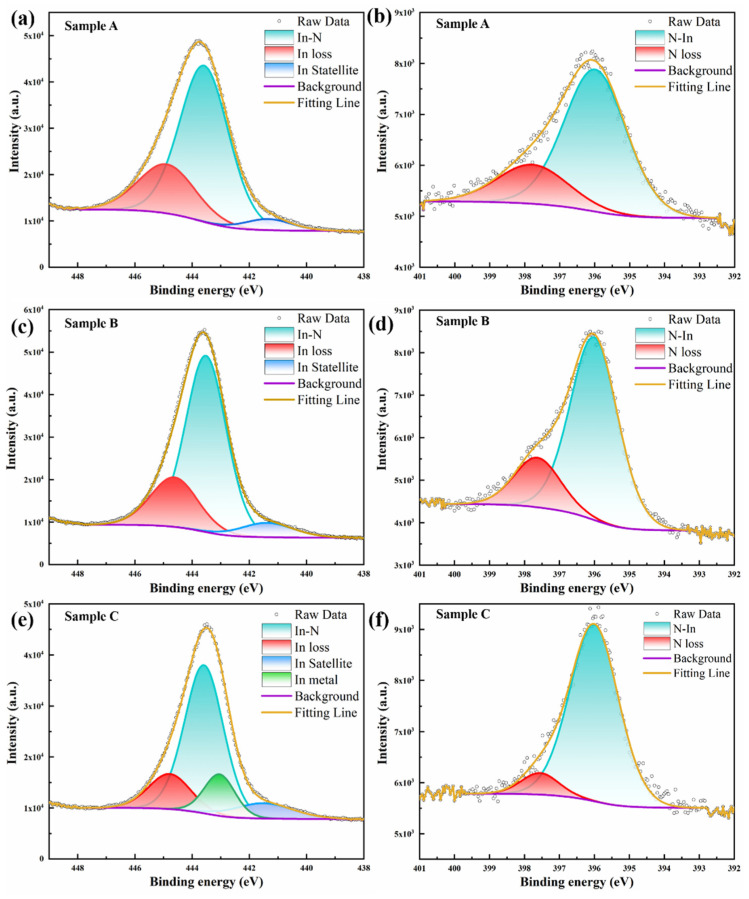
The In 3d_5/2_ (**a**,**c**,**e**) and N 1s (**b**,**d**,**f**) XPS core-level photoemission peaks of samples A, B, and C.

Figure 5 presents the XPS core-level peaks of In 3d_5/2_ and N 1s for samples A, B, and C. Sample A’s In 3d_5/2_ peak, as depicted in Figure 5a, exhibits distinct asymmetry, which is deconvoluted into three components. The less intense peak at 441.614 eV is attributed to the satellite peak from the X-ray source, the predominant peak at 443.597 eV corresponds to In-N chemical bonding, and the peak at 444.797 eV is associated with energy loss due to inelastic scattering of photoelectrons with accumulated electrons on the InN surface. The N 1s peak of sample A in Figure 5b is fitted with the N-In chemical bond peak at 396.007 eV and the energy loss peak at 397.554 eV. The positions of the In 3d_5/2_ and N 1s peaks obtained by our fitting are consistent with previously reported results [32]. Samples B’s In 3d_5/2_ and N 1s peaks, shown in Figurer 5c,d, are consistent with the components observed in sample A. Sample C’s In 3d_5/2_ peak, in addition to the three components seen in samples A and B, also includes a monomeric In component at 443.082 eV. This, in conjunction with the SEM and XRD analysis results, suggests the possible presence of a few nanometers of a monomeric In thin layer on the surface of sample C. The N 1s peaks in sample C have the same components as the other samples. Based on the fitting results, the In/N stoichiometric ratios for samples A, B, and C are calculated as 1.53, 1.37, and 1.12, respectively. Based on these results, this indicates that samples A and B, which exhibit larger lattice mismatches and employ the same growth mechanism, have higher indium fractions. It is hypothesized that larger lattice mismatches lead to a higher density of defects, facilitating the introduction of more In components near these defects during epitaxial growth. In contrast, the integrity of the crystal structure in the supersaturated precipitated InN grains of sample C makes it less likely to introduce non-stoichiometric In monomers, resulting in the presence of metallic In monomers at the top of the precipitated InN grains. Furthermore, the energy loss peak percentages for In are calculated to be 25.51%, 22.79%, and 15.66%, while those for N are 23.62%, 20.91%, and 13.51%, respectively. Consistent with the crystal quality analysis from XRD, InN samples with higher defect densities exhibit a stronger surface electron accumulation, suggesting that elevated defect densities increase electron concentration in epitaxial InN; this may also explain the hexagonal columnar morphology of sample C with the highest crystal quality in the SEM image.

**Figure 6:**
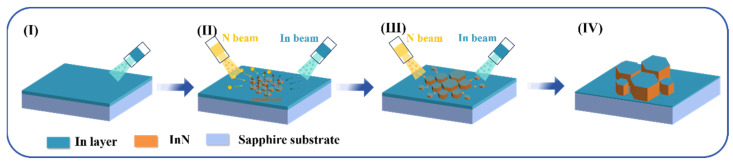
Growth mechanism schematic diagram of InN on sapphire with pre-deposited molten In layer.

Based on the experimental outcomes, the growth mechanism of InN crystals via the pre-deposited metallic In layer on sapphire can be delineated as follows (Figure 6): Initially, the In beam current is directed onto the heated sapphire substrate, forming a molten metal In layer (stage I). During stage II, the In and N beams irradiate the molten metal In layer, dissolving In and N atoms in the liquid-phase In layer until supersaturation is reached, triggering nucleation and the precipitation of InN, with excess metal In remaining on the top surface of the InN grains. Stage III is characterized by the spacing between small InN nuclei allowing for the dissolution of In and N atoms into the In layer, with epitaxy proceeding in an LPE mode. Finally, the increased size and density of InN nanopillars hinder the formation of new nucleation sites, leading to autocatalytic epitaxial growth predominantly along the c-axis orientation, utilizing the metal In on the top surface of the InN nanopillars as a catalyst, and culminating in the formation of hexagonal InN nanopillars with metallic In at the grain tops (stage IV).

**Figure 7:**
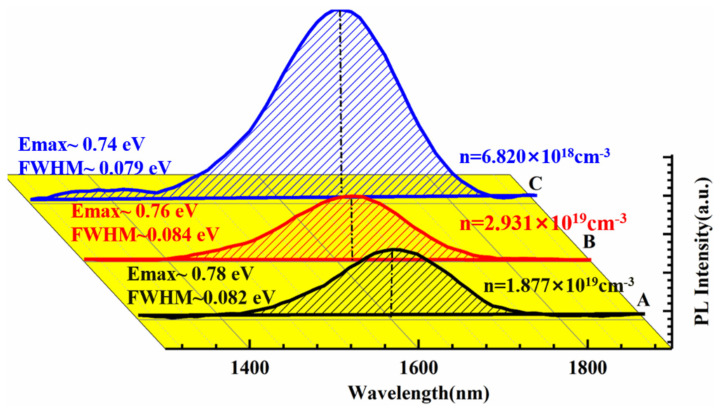
Room-temperature PL spectra of samples A, B, and C, alongside carrier concentrations derived from the Hall measurements.

**Figure 8:**
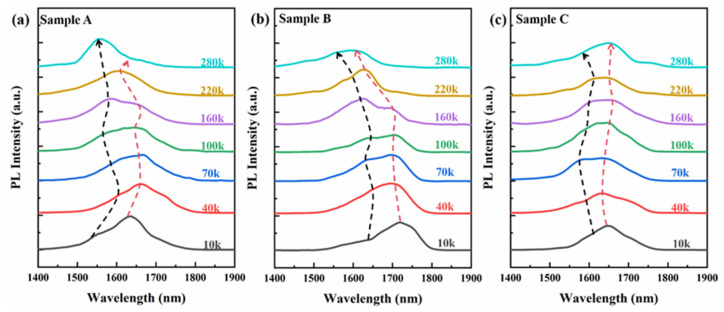
Temperature-dependent PL spectra of samples A (**a**), B (**b**), and C (**c**) at 10–280 k. The black dashed line shows the shift of the split peaks caused by the band-filling effect. The red dashed line is the split-peak shift caused by bandgap contraction.

We conducted room-temperature PL spectroscopy on the InN samples. As depicted in Figure 7, the centroid values of the luminescence peaks for samples A, B, and C are 0.78 eV, 0.76 eV, and 0.74 eV, respectively, with corresponding FWHMs of 0.079 eV, 0.087 eV, and 0.082 eV. Sample C, which employs a distinct growth mechanism, exhibits the most intense PL, the longest luminescence wavelength, and the narrowest PL FWHM compared to samples A and B, which are grown by the vapor-solid method. To study the complex factors influencing the luminescence properties of nanostructured InN, the Hall effect and temperature-dependent PL tests were performed on these samples, as shown in Figure 8. The Hall effect measurements reveal that all three samples possess a relatively high electron concentration, leading to the shift of the Fermi energy level into the conduction band and subsequently inducing the band-filling effect, which results in a blueshift and broadening of the InN PL peaks.

**Figure 9:**
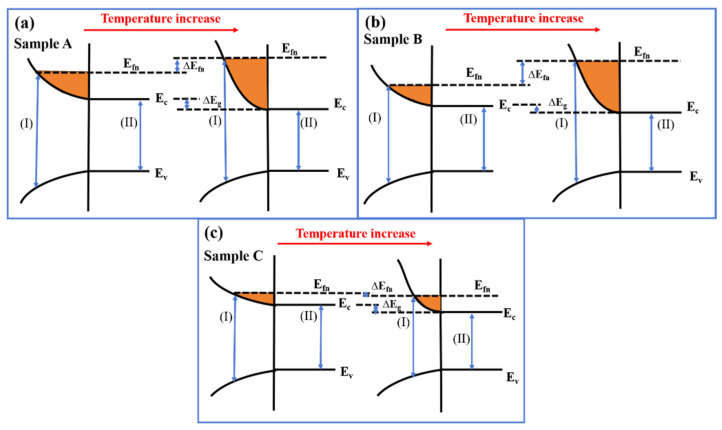
The energy band diagrams of samples A (**a**), B (**b**), and C (**c**) with the increase in temperature. (I) Band-filling effect. (II) Band-to-band radiative recombination.

As depicted in Figure 8a,b, the PL peak positions of samples A and B experience blueshifts of 33 nm and 105 nm, respectively, upon temperature elevation from 10 K to 300 K. Sample A, which exhibits a more pronounced shift to the high-energy end at 10 K, suggests a higher carrier concentration at low temperatures compared to sample B, and consequently, a more significant band-filling effect. With the onset of the temperature increase from 10 K, as illustrated in Figure 9a, the bandgap contraction due to thermal expansion leads to a redshift of the PL peak position for sample A. The higher carrier concentration in sample A at low temperatures intensifies the energy band-filling effect. As the temperature continues to rise, the blueshift effect from the band-filling effect becomes predominant, culminating in the final blueshift of the PL spectrum peak position for sample A. Sample B is subjected to the band-filling effect due to high-carrier concentration and, simultaneously, the SEM image reveals that sample B possesses a smaller wall width and higher surface-to-body ratio than sample A, thereby enhancing the strong localization effect of carriers [33], which further amplifies the band-filling effect. As shown in Figure 9b, this results in a continuous blueshift of the PL spectral peak position with increasing temperature and the most substantial change in the final wavelength blueshift. Sample C, as observed in Figure 8c, exhibits a subtle S-shaped fluctuation in the PL spectrum peak position with temperature increase, culminating in a mere 12 nm redshift, consistent with the low carrier concentration in sample C. According to the XPS analysis in Figure 5, sample C manifests the least surface electron accumulation among the trio. Consequently, we contend that this appearance is attributable to sample C’s hexagonal nanopillar structure, which is probably associated with a higher crystal quality, resulting in a lower carrier concentration, as well as a relatively low surface-to-body ratio. Evident in Figure 9c, it suggests subdued bandgap contraction and energy band-filling effects, which confer excellent temperature stability to its PL spectrum peak position.

## 4. Conclusions

In summary, the epitaxial growth of InN on large lattice-mismatched sapphire substrates via pre-deposited molten In layers by MBE facilitates the precipitation of InN grains through the LPE mechanism, and this approach is analyzed in comparison to the vapor-solid mechanism, a prevalent method in MBE. The SEM and XRD analysis show that the saturated crystals obtained from the pre-deposited molten In layer method exhibit a more complete crystal structure, significantly reducing defect density in epitaxial InN and facilitating the formation of high-quality, large-diameter hexagonal InN crystals with an intact lattice structure. The XPS analysis shows that this method effectively avoids introducing excess non-stoichiometric In monomers. In the vapor-solid growth method, the dependence of InN growth on substrate lattice matching, coupled with low growth temperatures and limited interatomic transverse migration, results in a reticulated morphology of the final InN. This approach is prone to generating high-density defects due to the stress imparted by the substrate during the growth process. Based on the Hall tests and PL spectroscopy, and compared to nano-networked InN grown on nitrided sapphire and GaN substrates by vapor-solid mechanism, the hexagonal nanopillar structure of InN obtained by this method exhibits a lower carrier concentration and weaker electron accumulation effect, thereby exhibiting excellent band-edge radiation composite efficiency and temperature stability of its luminescence wavelength. The high-performance InN synthesized via this method is anticipated to reduce reliance on refrigeration for high-power infrared optoelectronic device applications while enhancing luminescent performance, thereby advancing the development of optoelectronic devices.

## Figures and Tables

**Table 1:** Analysis of crystal properties of three InN samples.

Sample	Peak Position (°)	Lattice Constant c (nm)	FWHM (arcsec)	Dislocation Density (cm^−2^)
A	31.26	0.5715	7308	8.9 × 10^10^
B	31.33	0.5704	1620	4.3 × 10^9^
C	31.20	0.5725	684	7.8 × 10^8^

## Data Availability

The original contributions presented in this study are included in the article. Further inquiries can be directed to the corresponding author(s).
